# Gut microbiome signatures reflect different subtypes of irritable bowel syndrome

**DOI:** 10.1080/19490976.2022.2157697

**Published:** 2022-12-27

**Authors:** Qi Su, Hein M Tun, Qin Liu, Yun Kit Yeoh, Joyce Wing Yan Mak, Francis KL Chan, Siew C Ng

**Affiliations:** a Microbiota I-Center (MagIC), Hong Kong SAR, China; b Department of Medicine and Therapeutics, Institute of Digestive Disease, The Chinese University of Hong Kong, Hong Kong SAR, China; c The Jockey Club School of Public Health and Primary Care, Faculty of Medicine, The Chinese University of Hong Kong, Hong Kong SAR, China

**Keywords:** Irritable bowel syndrome, gut microbiome, subtype, diet, depression

## Abstract

Irritable bowel syndrome (IBS) is a heterogeneous condition with multifactorial pathogenesis. We studied deeply phenotyped individuals with microbiota sequencing enrolled in the American Gut Project. The IBS subjects were matched by age, gender, body mass index, geography, and dietary patterns with non-IBS controls. A total of 942 subjects with IBS-Diarrhea (IBS-D), IBS-Constipation (IBS-C), unclassified IBS (IBS-U), and 942 non-IBS controls were included. We compared taxonomic and functional composition of gut microbiota based on 16S sequencing data and linked them with clinical characteristics and dietary factors. Subjects with IBS-D or IBS-U but not IBS-C showed significantly reduced bacterial diversity (Shannon; p < .01). Distinct bacterial signatures were associated with different IBS subtypes, and the related functional changes were related to IBS pathogenesis, such as the increased hydrogen sulfide production pathway in IBS-D and the increased palmitoleate biosynthesis pathway in IBS-C. IBS subjects with depression showed lower abundance of *Bifidobacterium, Sutterella, Butyricimonas* and higher abundance of *Proteus* than those without depression. The relative abundance of microbial short-chain fatty acid production pathways was significantly lower in IBS patients with depression than those without depression in all three subtypes. Female, younger age in IBS-D, and older age in IBS-C were associated with more severe microbiota dysbiosis, and distinct dietary factors had significant effects on the gut microbiota in different IBS subtypes. Our analysis identified the compositional uniqueness of gut microbiota in different IBS subtypes. Distinct associations of the gut microbiota with depression in IBS provide insights into shared pathways in disease pathogenesis. These findings highlight the importance of personalized gut microbiome modulation approaches in different subtypes for optimal therapeutic effects.

## Introduction

Altered gut microbiome has been reported to play a role in the pathophysiology of irritable bowel syndrome (IBS), and accumulating data reported that specific bacteria were either enriched or depleted in the gut of patients with IBS.^1^ However, IBS is a heterogeneous condition with different subtypes and varying symptoms, but whether gut microbiota composition varies with different subtypes of IBS has not been studied.^2,3^ Psychosocial factors have been linked to a worse prognosis in IBS,^4^ in particular, a higher prevalence of depression has been reported in patients with IBS,^5^ suggesting that altered brain-gut axis may in part play a role in the pathogenesis of depression in IBS.^6^ A previous study has reported correlation between diet and gut microbiome composition in patients with IBS.^7^ Furthermore, over two-thirds of IBS patients reported that their symptoms were triggered by dietary factors,^8^ while specific dietary interventions, such as the low FODMAP diet,^9^ have been shown to relieve IBS by effectively modulating the gut microbiota.^10^

Identifying associations between clinical phenotypes of IBS and gut microbiota as well as dietary factors may facilitate more personalized therapeutic targets. Herein, we examined the gut microbiota compositions of 942 comprehensively phenotyped patients with IBS based on sequencing data from the American Gut Project (AGP)^11^ using a one-to-one pairing algorithm with an equal number of matched controls.^12^

## Results

### Cohort characteristics

**Figure 1.**
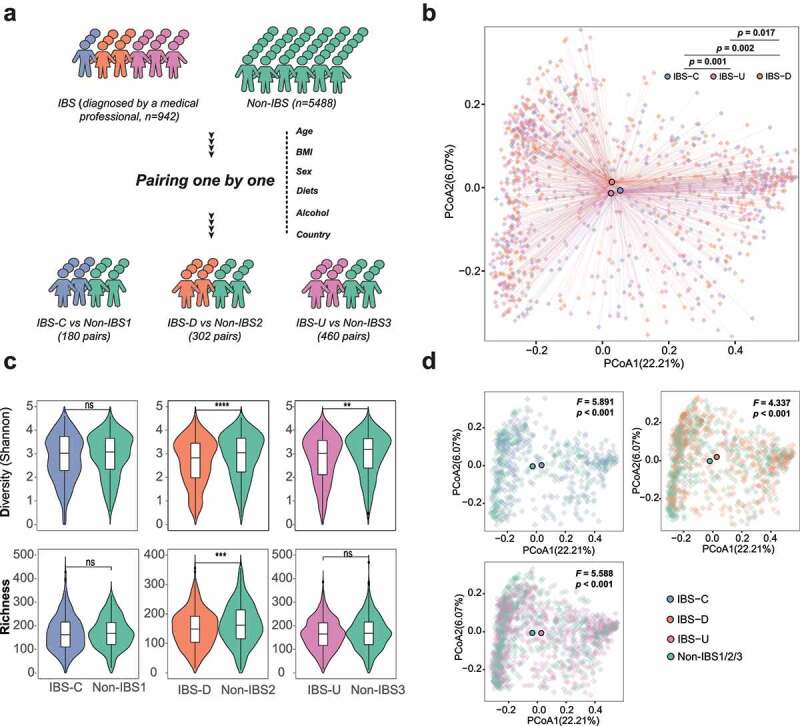
Cohort construction and microbiota characteristics of three IBS subtypes. (a) Conceptual outline of cohort construction and classification; (b) Principal Coordinates Analysis of gut microbiota composition of patients with different IBS subtypes by Bray-Curtis dissimilarities, the difference among IBS subtypes was assess by PERMANOVA test. (c) Diversity (Shannon Index) and richness of gut microbiota of IBS patients and non-IBS controls in different subtypes, * p < .05, ** p < .01, *** p < .001, **** p < .0001; (d) Principal Coordinates Analysis of gut microbiota composition of patients with different IBS subtypes and corresponding non-IBS controls by Bray-Curtis dissimilarities, the difference among IBS subtypes was assess by PERMANOVA test.

A total of 942 IBS patients with detailed clinical phenotype data and dietary report (Supplementary Table 1) were paired with 942 non-IBS subjects who were matched by age, BMI, country, diet (including alcohol), and gender, resulting in a total of 1884 subjects (Supplementary Figure 1, Figure 1a), which was further divided into three sub-cohorts based on stool consistency,^13^ namely IBS-C vs non-IBS1 (180 pairs), IBS-D vs non-IBS2 (302 pairs), and IBS-U vs non-IBS3 (460 pairs). There was no difference in BMI, sex, age, geographical location, alcohol consumption, and frequency of dietary intake of meat/eggs, dairy, vegetables, whole grain, and salted snacks between the patients with IBS and their paired controls (p > .05, Table 1, Supplementary Figure 2). There was also no difference in age, sex, BMI, geographical location, and diets among different subtypes of patients with IBS (Supplementary Table 2).

**Table 1.** Demographics of involved IBS patients and non-IBS controls.

Demographics	IBS patients (N = 942)	non-IBS controls (n = 942)	*p* Value
Age (median, IQR)	47 (36–58)	49 (37–65)	0.063
Female (n, %)	618 (65.5%)	618 (65.5%)	1
BMI (median, IQR)	24 (21–26)	24 (21–26)	0.877
USA (n, %)	470 (49.9%)	470 (49.9%)	1
UK (n, %)	463 (49.1%)	463 (49.1%)	1
Canada (n, %)	9 (0.9%)	9 (0.9%)	1

### Gut microbiota signatures in different subtypes of IBS

Based on principal coordinates analysis (PCoA) of Bray-Curtis dissimilarities, patients with different subtypes of IBS including IBS-C, IBS-D, and IBS-U showed distinct clustering of their gut microbiota composition (p < .05, PERMANOVA test, Figure 1b). Gut microbiota composition of these three IBS subtypes was distinct from that of controls (p < .001. PERMANOVA test, Figure 1d). Bacteria diversity was significantly reduced in subjects with IBS-D and IBS-U (p < .0001, p < .01, paired samples t-test, Figure 1c) but not in subjects with IBS-C (p > .05, paired samples t-test, Figure 1c) when compared with their respective controls. Subjects with IBS-D had a significant decrease in bacteria richness compared to controls (p < .001, paired samples t-test, Figure 1c). No difference between alpha/beta-diversity and richness was found among the three control groups (Supplementary Figure 3).

**Figure 2.**
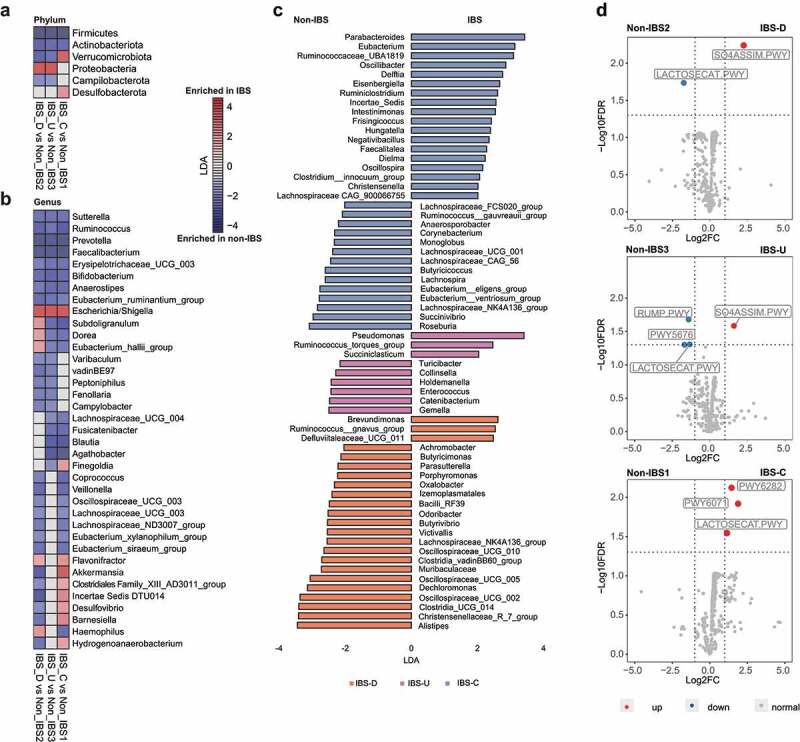
Compositional and functional alterations in gut microbiota of patients with different IBS subtypes. (a) The alterations of six phyla in different IBS subtypes by comparing to corresponding non-IBS control; (b) The alterations of 37 genera in different IBS subtypes by comparing to corresponding non-IBS control; (c) 64 microbial genera that only altered in one specific IBS subtype. The alteration was assessed by comparing IBS cohort and non-IBS cohort for each subtype using the linear discriminative analysis (LDA) effect size biomarker discovery tool (p < .05, LDA >2, FDR<0.1). (d) Volcano plots demonstrating bacterial MetaCyc pathway alterations in IBS-D, IBS-U and IBS-C; A binomial test was used to calculate the p values for the up-regulation or down-regulation of MetaCyc pathway in the IBS cohort versus the non-IBS cohort for each subtype. Colored dot indicate pathway with FDR- adjusted p < .05.

Since confounding factors were fully considered when constructing cohort, we performed the linear discriminative analysis (LDA) effect size (LEfSe) analysis for IBS-C, IBS-D, and IBS-U independently with the corresponding paired control group. Six bacterial phyla were identified to be associated with different IBS subtypes (p < .05, LDA > 2, FDR < 0.1, LEfSe, Figure 2a, Supplementary Tables 3–5). At the phylum level, subjects with IBS-D and IBS-U shared similar compositional alterations driven by depletion of *Firmicutes, Actinobacteriota, Verrucomicrobiota, Campilobacterota*, and enrichment of *Proteobacteria* compared with controls. Subjects with IBS-C showed increased abundance of *Verrucomicrobiota*, and *Desulfobacterota* (Figure 2a). A total of 101 bacteria genera were identified to be associated with different IBS subtypes (Figure 2b,c, Supplementary Table 6). Nine bacterial genera including *Sutterella, Faecalibacterium, Bifidobacterium* were found to be significantly decreased in all three IBS subtypes (p < .05, LDA > 2, FDR < 0.1, Figure 2b). In contrast, the pathogenic bacteria *Escherichia/Shigella*^14,15^ were found to be significantly increased in all three IBS subtypes (p < .05, LDA > 2, FDR < 0.1, Figure 2b). Eleven bacterial genera showed opposite trends across different IBS-subtypes (Figure 2b). For example, *Subdoligranulum*, *Dorea*, *Eubacterium hallii* and *Haemophilus* were enriched in subjects with IBS-D but depleted in IBS-C. We found that half (64 of 101) of the bacterial taxa only differed in one of the three IBS subtypes (Figure 2c). Many of the bacteria found to be enriched in IBS were opportunistic pathogens, including *Ruminococcus gnavus* in IBS-D, *Ruminococcus torques* in IBS-U, and *Oscillibacter* in IBS-C (Figure 2c). In contrast, several beneficial bacteria were depleted in different subtypes of IBS, for example *Butyricoccus* in IBS-C, *Turicibacter* in IBS-U, and *Alistipes* in IBS-D.

When all subtypes were put in the same group, bacterial diversity of patients with IBS was lower than that of non-IBS controls but no difference in richness (Supplementary Figure 4A, B). The difference in gut microbiota composition between mixed IBS and non-IBS tested by PERMANOVA (F = 1.43, p = .045, Supplementary Figure 4C) was smaller than that in subtype level (5.891 for IBS-C vs non-IBS1, 4.337 for IBS-D vs non-IBS2, and 5.588 for IBS-U vs non-IBS3, p < .001, Figure 1d). No functional difference was observed between the mixed IBS and non-IBS controls (FDR <0.05, Supplementary Figure 4D). Only 56 bacteria genera showed significant alternation in comparing mixed IBS and non-IBS controls (p < .05, LDA >2, FDR < 0.1, Supplementary Figure 4E, Supplementary Table 7), demonstrating incomplete subtype-specific microbiota signatures in unsubtyped analysis (Supplementary Figure 4 F), particularly some genera with opposite alteration trends in different subtypes like *Akkermansia* (decreased in IBS-D but increased in IBS-C, Figure 2).

Data released by AGP from Apr-2021 to Feb-2022 were used for constructing a validation cohort. Following the same process employed in this study, a total of 153 pairs of IBS (IBS-D, n = 48; IBS-U, n = 61; IBS-C, n = 44) and non-IBS controls were selected and matched from 4974 controls (Supplementary Figure 5A). Principal coordinates analysis (PCoA) based on Bray-Curtis dissimilarities verified the difference in beta-diversity among different IBS subtypes (Supplementary Figure 5B). Again, we observed the significant decrease in alpha-diversity in IBS-D and IBS-U patients and the reduced richness in IBS-D patients (Supplementary Figure 5C). The LEfSe analysis at phylum and genus level also confirmed the distinct gut microbiome signatures in different IBS subtypes (Supplementary Figure 5C).

### Functional changes of the gut microbiota in different IBS subtypes

In stool samples from different subtypes, 6 out of 481 identified microbial metabolic pathways showed significant alteration compared with paired controls (2 in IBS-D, 4 in IBS-U, 3 in IBS-C, respectively; FDR <0.05; Figure 2d). Specifically, the LACTOSECAT pathway involved in the degradation of lactose and galactose was decreased in IBS-D and IBS-U, but increased in IBS-C. An increment of SO4ASSIM pathway, which can produce hydrogen sulfide, was found in both IBS-D and IBS-U. The butyrate production pathway of acetyl-CoA fermentation (PWY5676) as well as the formaldehyde oxidation pathway (RUMP pathway) were both decreased in IBS-U. In addition, two other pathways related to palmitoleate biosynthesis (PWY6282) and phenylethylamine degradation (PWY6071) were only elevated in IBS-C. Spearman correlation analysis was used to show the associations between these functional pathways and gut microbiota in different subtypes (Supplementary Figure 6). Briefly, in IBS-D, *Escherichia/Shigella*, which was found to be increased in this subtype, exhibited the strongest positive correlation with hydrogen sulfide production (SO4ASSIM pathway, R = 0.46, p < .0001) and the strongest negative correlation with the degradation of lactose and galactose (LACTOSECAT pathway, R = 0.23, p < .0001). These results implied that the specific microbiome alteration in each IBS subtypes further lead to distinct functional changes.

### Gut microbiota alterations and functional changes in IBS patients with depression

**Figure 3.**
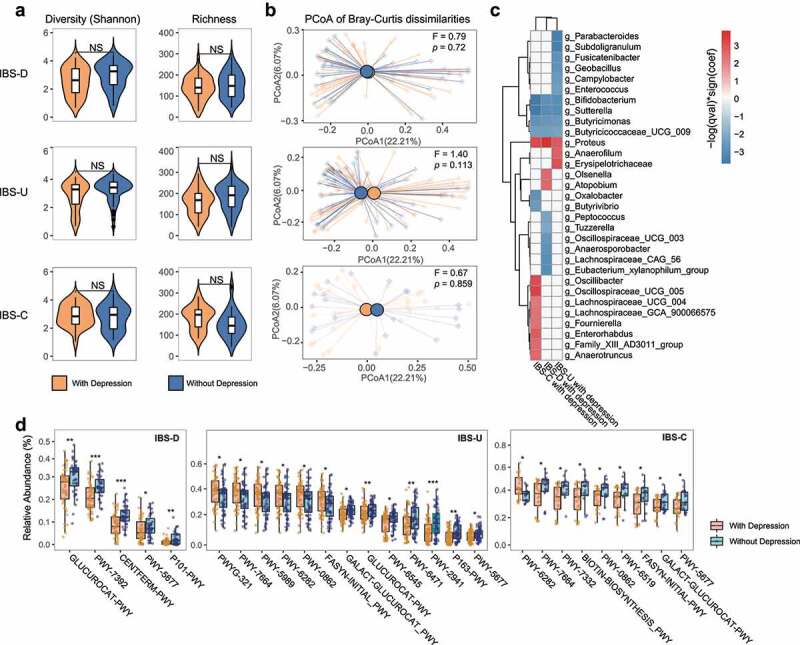
Bacterial and functional alterations in gut microbiota of IBS patients with depression. (a) Diversity (Shannon Index) and richness of gut microbiota of IBS patients with depression or without depression in different subtypes, NS means not significant. (b) Principal Coordinates Analysis of gut microbiota composition of IBS patients with depression corresponding IBS patients without depression in different subtypes by Bray-Curtis dissimilarities, the difference among IBS subtypes was assessed by PERMANOVA test. (c) The association between gut microbiota and IBS patients with depression in different subtypes. The association was assessed by the multivariate analysis by linear models (MaAsLin; p < .05, FDR<0.1). (d) MetaCyc pathway differences between IBS patients with depression and those without depression, * p < .05, ** p < .01, *** p < .001, **** p < .0001. In each subtype, an equal number of IBS patients without depression were selected using the established matching algorithm.

**Figure 4.**
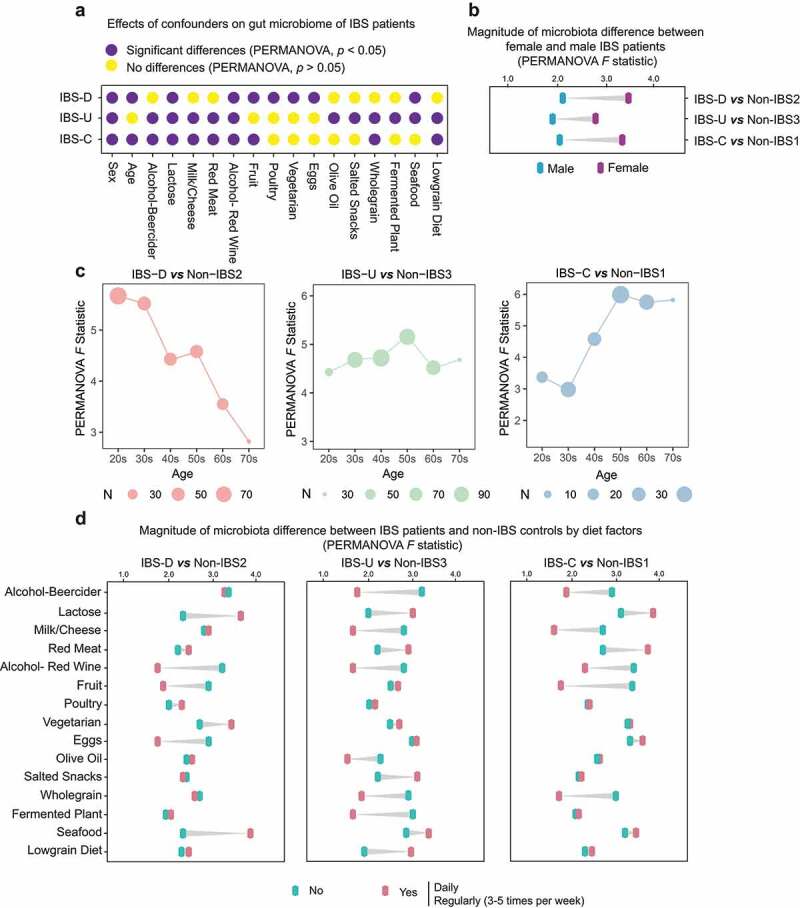
Effects of age, gender and dietary factors of the gut microbiota in IBS patients. (a) The effects of host confounders on gut microbiome of IBS patients (PERMANOVA, p < .05), IBS means all patients without subtyping. (b) Differences between beta-diversity-based *F* statistic of IBS patients and non-IBS controls grouped by gender in different subtypes. (c) Differences between beta-diversity-based *F* statistic of IBS patients and non-IBS controls grouped by age in different subtypes. (d) Differences between beta-diversity-based *F* statistic of IBS patients and non-IBS controls grouped by diets in different subtypes. “No” means never eat the specific food, and “Yes” means eating this kind of food at a high frequency (daily or regularly).

A subset of patients with IBS had a diagnosis of depression (IBS-D, n = 36; IBS-U, n = 63; IBS-C, n = 35). We paired patients with IBS on depression, with others without depression using the above matching algorithm adjusting for age, BMI, country, diet (including alcohol), and sex. There was no difference in alpha diversity (Shannon Index), richness, and the composition structure (PCoA based on Bray-Curtis dissimilarities) between IBS patients with depression and those without depression in all three subtypes (p > .05, Figure 3a,b). We found that IBS patients with depression in all three subtypes were negatively associated with the beneficial bacteria *Bifidobacterium, Sutterella, Butyricimonas, Butyricicoccaceae UCG009*, but positively associated with *Proteus* (p < .05, FDR <0.1, MaAsLin2, Figure 3c). A total of 20 MetaCyc pathways were identified to be associated with IBS patients with depression versus IBS patients without depression (p < .05, FDR < 0.05, Figure 4d, Supplementary Table 9), including the depletion of several pathways involving in the synthesis of short chain fatty acids (SCFAs) such as pyruvate fermentation to butanoate (CENTFERM-PWY), L-lysine fermentation to acetate and butanoate (P163-PWY) and succinate fermentation to butanoate (PWY-5677). Moreover, a superpathway of β-D-glucuronide and D-glucuronate degradation (GLUCUROCAT-PWY) decreased in all three subtypes. We also correlated the relative abundance of *Bifidobacterium, Sutterella, Butyricimonas, Butyricicoccaceae UCG009*, and *Proteus* with these pathways (FDR <0.1, Spearman correlation, Supplementary Table 8), which further illustrated the relationship between bacteria and function.

### Effects of sex and age on the gut microbiome of patients with IBS

In this cohort, females account for more than 62% of IBS patients in all three subtypes (Supplementary Table 2). The differences between female and male IBS patients were significant in all subtypes (p < .05, PERMANOVA test, Figure 4a). The gut microbiomes of female IBS patients in all three subtypes showed greater alterations (F = 2.8 ~ 3.5) than male (F = 1.8 ~ 2.1) when compared to corresponding paired controls of the same gender (Figure 4b). Besides, the median age of IBS-C patients (51, IQR 36–59) and IBS-U patients (48, IQR 33–59) was older than that of IBS-D patients (37, IQR 25–48, Supplementary Table 2). We observed that age showed a significant effect on the gut microbiome of IBS-D and IBS-C patients (Figure 4a). The magnitude of the microbiota difference between IBS patients and non-IBS controls decreased with age in IBS-D but increased in IBS-C (PERMANOVA *F* pseudo statistic, Figure 4c).

### Effects of dietary factors on the gut microbiome of patients with IBS

We found that a series of dietary factors such as alcohol, lactose, milk, etc., exhibited significant associations on the gut microbiota of IBS patients (27 foods were analyzed, Supplementary Table 1) and these associations vary with subtypes (p < .05, FDR < 0.1, PERMANOVA test, Figure 4a). Among them, lactose and red wine had a significant effect on the gut microbiota of IBS patients across all subtypes (Figure 4a). The PERMANOVA pseudo ***F*** statistic was employed to further show the associations of dietary factors on the gut microbiota of IBS patients. In all three subtypes, subjects who took lactose daily or regularly (3–5 times per week) exhibited larger microbiome alterations than subjects who never had lactose intake (IBS patients versus paired controls divided by lactose intake), while those who drank red wine achieved the opposite effect (Figure 4d). Several foods narrowed the microbiota difference between IBS patients and controls, such as milk/cheese or whole grain for IBS-U and IBS-C, fruit for IBS-D and IBS-C, as well as eggs for IBS-D, while others like seafood for IBS-D enlarged the difference (Figure 4d).

## Discussion

In this study, we established a fully phenotyped IBS-control cohort (n = 1884) where all the selected cases and controls were strictly filtered and paired from AGP of over 27,000 deeply phenotyped participants,^11,12^ which properly addressed the problems of small sample sizes, wide interindividual heterogeneity, and unbalanced confounding host variables.^1^ Through this cohort, we identified 101 subtype-specific gut microbiome signatures, determined microbiological factors associated with concomitant depression, explored the associations between gut microbiome and age, sex, and diet in IBS patients, providing insights into the heterogeneity and multifactorial pathogenesis.

This study identified bacteria related to the clinical phenotype of IBS. For example, *Ruminococcus gnavus*, which can induces inflammatory cytokine (TNFα) secretion,^16^ was more abundant in IBS-D, while constipation-related *Oscillospira*^17^ were enriched in IBS-C. We also identified several shared microbial signatures across subtypes, such as *Sutterella*, which provide new targets for developing general anti-IBS interventions. The 101 subtype-specific bacterial signatures also provided a pool of probiotic candidates against specific IBS subtypes, such as *Akkermansia*, which may alleviate IBS-D.

Subtype-specific microbiota signatures are also reflected in functional alterations. SO4ASSIM pathway that can produce hydrogen sulfide was enhanced in IBS-D and IBS-U. Hydrogen sulfide has antioxidant and immune-regulatory properties, and it can also serve as an oral gas biomarker for IBS-D,^18^ while our findings explain that the accumulated hydrogen sulfide is from the increased SO4ASSIM pathway. Some studies have directly confirmed that hydrogen sulfide can induce diarrhea,^19^ thus it may also be responsible for the altered stool quality in IBS-D patients. In addition, the LACTOSECAT pathway (increased in IBS-C but decreased in IBS-D and IBS-U) is mainly involved in the degradation of lactose and galactose, and a decrease in this function appears to cause lactose-related dyspepsia, which is an important trigger for IBS symptoms.^20,21^ More importantly, our results also revealed that avoiding lactose intake narrows the differences in gut microbiota between IBS patients and controls. All of these provided clues for understanding the effect and mechanism of lactose-free diet on IBS patients. Another novel finding was that the pathway of palmitoleate biosynthesis was increased in IBS-C. While the product of this pathway is palmitic acid, which tends to react with calcium to form insoluble calcium palmitate, and the level of this calcium palmitate is positively correlated with stool hardness.^22^ Therefore, the enhanced palmitoleate biosynthesis might be the reason for the constipation in IBS-C patients.

Depression is often associated with IBS,^23^ but the exact pathophysiology underlying this remains unclear.^24^ Our results showed that IBS patients with depression had a significantly lower abundance of beneficial bacteria such as *Bifidobacterium*^25^ and higher levels of pathogenic bacteria including *Proteus^26^* than those without depression. *Proteus* has been proven to damage neurons in mice,^27,28^ so its enrichment in IBS patients with depression may be an important pathogenic factor. In addition, several functional pathways related to SCFAs production were less abundant in IBS patients with depression compared to those without depression. Previous studies have reported reduced concentrations of SCFAs in depressed mice and humans,^29,30^ thus lower levels of these pathways may also lead to reduced levels of SCFAs in IBS patients, which may then induce depression. Bacteria depleted in IBS patients with depression like *Bifidobacterium* are the main strain producing SCFAs, thus supplementing these beneficial bacteria may alleviate the symptoms.

The most important risk factors for IBS are female sex and younger age,^4,31^ while our results show that the microbiota compositional difference between female IBS patients and controls is larger than that of males, which suggests that females are more prone to not only IBS but also more severe gut dysbiosis. We also observed an effect of age on the gut microbiota of IBS patients. The magnitude of microbiota compositional difference between IBS patients and non-IBS controls becomes smaller with age in IBS-D but larger in IBS-C, which may explain why young people are prone to IBS-D and older people are prone to IBS-C.^2^ Diet plays an important role in regulating the gut microbiome.^32^ Our results show that the effect of dietary factors on gut microbiota varies with IBS subtypes, hence targeted dietary intervention such as eating more fruit or avoiding lactose may help different IBS patients alleviate symptoms.

This study established the largest deeply phenotyped IBS-control cohort, accurately depicted the gut microbiome signatures at subtype level, and further linked them to depression, age, sex, and diet, which has certain implications for the pathogenesis of IBS. The main findings were also verified by an independent cohort, which further illustrated the robustness of the results. There are also some limitations. This is an observational cross-sectional analysis and some residual and potential confounders maybe not accounted for, and there may be recall bias since diet data is captured using questionnaires. Also, the phenotype data were collected by questionnaire, there may be unknown bias for subtype classification. Moreover, due to the lack of accurate data to meet the criteria of IBS-M, this subtype was not included in our study, and future studies should focus on it to fully understand the association between microbiota and subtypes.

In conclusion, our results uncover new insights related to distinct gut microbiome compositional and functional signatures in different subtypes of IBS. These findings highlight the importance of personalized gut microbiome modulation approaches in different subtypes for optimal therapeutic effects.

## Methods

**AGP and data availability** The AGP was launched by the American Gut Consortium since November 2012.^11^ Detailed recruitment process for participants is reported on http://americangut.org. A full list of the involved host variables is summarized in Supplementary Table 1. Details about sample collection and processing have been published previously.^11^ Briefly, samples were collected using BBL culture swabs (Becton, Dickinson and Company, Sparks, MD), returned by mail, and stored at −80°C. All samples were processed using the EMP protocols. The DNA of samples was extracted for 16 rRNA sequencing (V4 region) with an Illumina HiSeq Rapid Run.^11^ Sequence data are available from the European Bioinformatics Institute (EBI) database under study accession ID: MGYS00000596. Participants’ consents were obtained under the Institutional Review Board human research subject protocols from the University of Colorado, Boulder (protocol no. 12–0582; December 2012 to March 2015) or from the University of California, San Diego (protocol no. 141853; February 2015 to present). No personally identifiable data was included in the public database nor was accessed in the present study.

### Building of fully paired cohorts for IBS and non-IBS controls

Detailed process about the cohort construction can be found in Supplementary Figure 1. Briefly, 27206 records were downloaded from EBI in Apr 2021, and 3137 of them had IBS symptoms. Self-diagnosed cases of IBS or those diagnosed by an alternative medicine practitioner were excluded, and a total of 2,004 patients diagnosed by a medical professional [doctor, physician assistant] were eligible. According to a previous study that evaluated the effects of host factors on gut microbiota,^12^ the IBS group were further narrowed into 942 subjects by the following exclusion criteria: younger than 18 or older than 80; BMI less than 12.5 or greater than 40; diagnosed with inflammatory bowel disease (IBD) or Type II Diabetes; live outside the United States, the United Kingdom, and Canada; no fecal samples were provided; bowel movement quality was not available; took antibiotics within 6 months. Controls consisted of 5,488 subjects who were selected under the same criterion from a pool of 12,915 subjects who did not have symptoms of IBS.

The pairing algorithm was constructed based on previously identified microbiota-confounding variables,^12^ including BMI, sex, age, geographical location, alcohol consumption frequency, and dietary intake frequency of meat/eggs, dairy, vegetables, whole grain, and salted snacks. The pairwise Euclidean distances were computed between the IBS and control groups from the above set of matching variables that were normalized to zero-mean and unit variance (centered and scaled). Subsequently, an IBS sample and the closest control sample were removed from the selection group and then added to the cohort. The selection process is successive until no IBS samples remain in the selection group. If multiple control samples share the closest distance with IBS sample, the pairwise Euclidean distances among them will be re-calculated based on other host variables (Supplementary Table 1).

Then, the paired case–control cohorts were further divided into sub-cohorts of different IBS subtypes according to the ROME Ⅳ criteria^2^ based on phenotype data. Specifically, the clinical definition of IBS-D was defined as “I tend to have diarrhea (watery stool)”, and the definition of IBS-C was classified as “I tend to be constipated (have difficulty passing stool)”. Because there were insufficient precise and clear data to meet the criteria for IBS-M that more than 25% of bowel movements with Bristol Stool Score (BSS) 1 or 2 and more than 25% of bowel movements with BSS 6 or 7, thus our analysis did not include the subtype of IBS-M to avoid less robust results. The IBS-U is a distinct category in this study and was classified as subjects who reported to have normal formed stool (“I tend to have normal formed stool”). Finally, three sub-cohorts were constructed, including IBS-D and their paired controls (non-IBS2), IBS-C, and their paired controls (non-IBS1), as well as IBS-U and their paired controls (non-IBS3). Data released by AGP from Apr-2021 to Dec-2022 were used for constructing a validation cohort using the similar process.

### Metadata curation and sub-cohort construction

Metadata with a range of host variables covering clinical characteristics, lifestyle, and diet factors for all participants in AGP were downloaded from the EBI. The majority of host variables in the metadata can be defined as binary with a positive and negative class, which allowed for simple construction of a subset of the samples to be used for binary comparison. Frequency variables were present in the metadata for a set of standard foods and beverage types, in which participants recorded their consumption frequency from a set of five values: daily, regularly (3–5 times per week), occasionally (1–2 times per week), rarely (less than once a week) and never. Binary matched cohorts were constructed from diet frequency variables in which participants who belonged to the ”daily” and ”regularly” consumption groups were matched with the set of participants that belonged to the ”never” group, which was taken as the negative class, and the participants in group ”occasionally” and ”rarely” were excluded to reduce the error rate. Only participants who answered “female” or “male” were reserved for the construction of binary matched cohorts concerning gender. All binary sub-cohorts have a balanced number through the above-mentioned matching algorithm.

### Processing of 16s rDNA sequence data

Raw fastq files were downloaded from the EBI and processed using the QIIME2.^33^ First, samples with fewer than 10,000 reads were removed from the analysis. Reads were denoised by the embedded DADA2^34^ for trimming with a maximum expected error threshold of 1, and reads were truncated at the first base with a Q score of 11 or below. Considering the importance of high-quality sample processing to the results of microbiome profiling,^35^ previously recognized sequences that were prone to blooming in the process of transporting fecal samples at room temperature were removed by Deblur.^11,36^ A table of 16S rRNA amplicon sequence variants (ASVs) was generated, and then annotated using the silva-132-99-515-806-nb-classifier.^33,34^ Sequence variants that were present in fewer than 50 samples and in lower total relative abundance than 0.01% were removed from the feature table. The feature tables at different taxa levels (phylum to species) were obtained from QIIME2 online tool (https://view.qiime2.org/). PICRUSt2 (version alpha.2) was used to generate a function prediction for MetaCyc metabolic pathways.^37^

### Statistical analysis

All analyses were carried out in R software V4.0.5. Principal coordinate analysis (PCoA) was used to visualize the clustering of samples based on their bacterial relative abundance, and differences in community composition were tested using paired sample t-test and permutational multivariate analysis of variance (PERMANOVA) with FDR correction. Associations of specific microbial taxa in different levels with participant parameters were identified using the linear discriminant analysis effect size (LEfSe) since we strictly matched the confounding factors of case and control. The multivariate analysis by linear models (MaAsLin2) statistical frameworks was implemented in the Huttenhower Lab Galaxy instance (http://huttenhower.sph.harvard.edu/galaxy/). Alpha-Diversity (diversity within samples) was assessed using the number of observed species rarefied at the same sequencing depth (10000 sequences per sample in this study). Microbiota compositional characteristics (abundance, diversity, and richness), PCoA, PERMANOVA, and Procrustes analysis were implemented in the vegan R package V.2.5–7. Data were visualized with pheatmap, ggplot2, and dependent R packages. All authors had access to the study data and reviewed and approved the final manuscript.

## Supplementary Material

Supplemental Material

## Data Availability

Sequences and metadata are available from the European Bioinformatics Institute (EBI) database under project ID: PRJEB11419 (https://www.ebi.ac.uk/ena/browser/view/PRJEB11419).

## Code availability

Source code for the case–control matching algorithms was from a previous study^12^. Code for statistical analyses can be obtained by reasonable request.
