# Supplementary material for: Gut microbiome signatures reflect different subtypes of irritable bowel syndrome
Source: Gut Microbes. 2022 Dec 27;15(1):2157697. doi: 10.1080/19490976.2022.2157697 (PMC9809927; doi:10.1080/19490976.2022.2157697)
Supplement: Supplemental Material [file KGMI_A_2157697_SM8569.zip › Supplementary Figure 4.pdf]

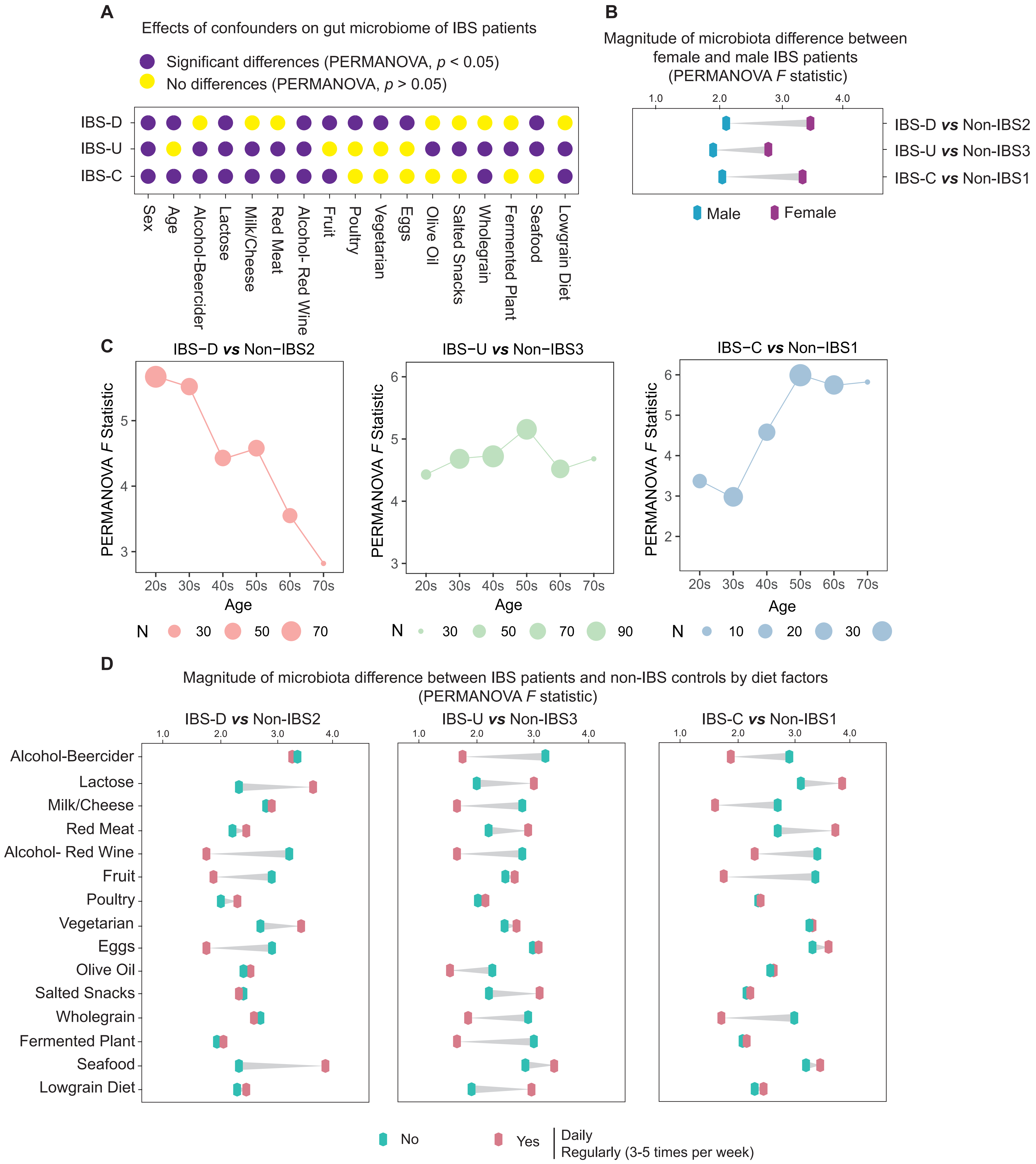
A
Effects of confounders on gut microbiome of IBS patients
Significant differences (PERMANOVA, p < 0.05)
No differences (PERMANOVA, p > 0.05)
IBS-D
IBS-U
IBS-C
Sex
Age
Alcohol-Beercider
Lactose
Milk/Cheese
Red Meat
Alcohol- Red Wine
Fruit
Poultry
Vegetarian
Eggs
Olive Oil
Salted Snacks
Wholegrain
Fermented Plant
Seafood
Lowgrain Diet
B
Magnitude of microbiota difference between female and male IBS patients (PERMANOVA F statistic)
1.0
2.0
3.0
4.0
IBS-D vs Non-IBS2
IBS-U vs Non-IBS3
IBS-C vs Non-IBS1
Male
Female
C
IBS−D vs Non−IBS2
IBS−U vs Non−IBS3
IBS−C vs Non−IBS1
PERMANOVA F Statistic
Age
20s 30s 40s 50s 60s 70s
N 30 50 70
N 30 50 70 90
N 10 20 30
D
Magnitude of microbiota difference between IBS patients and non-IBS controls by diet factors (PERMANOVA F statistic)
IBS-D vs Non-IBS2
IBS-U vs Non-IBS3
IBS-C vs Non-IBS1
1.0
2.0
3.0
4.0
Alcohol-Beercider
Lactose
Milk/Cheese
Red Meat
Alcohol- Red Wine
Fruit
Poultry
Vegetarian
Eggs
Olive Oil
Salted Snacks
Wholegrain
Fermented Plant
Seafood
Lowgrain Diet
No
Yes
Daily
Regularly (3-5 times per week)
