# Supplementary material for: Gut microbiome signatures reflect different subtypes of irritable bowel syndrome
Source: Gut Microbes. 2022 Dec 27;15(1):2157697. doi: 10.1080/19490976.2022.2157697 (PMC9809927; doi:10.1080/19490976.2022.2157697)
Supplement: Supplemental Material [file KGMI_A_2157697_SM8569.zip › Supplementary Table 2.docx]

**Supplementary Table 2** Demographics of IBS patients in different subtypes

| Demographics | IBS-D (N=302) | IBS-U (N=460) | IBS-C (N=180) |
| --- | --- | --- | --- |
| Age (median, IQR) | 36.7 (25 -48) | 48.3 (33 - 59) | 51.0 (36 - 59) |
| Female (n, %) | 188 (62.2%) | 287 (62.5%) | 143 (79.4%) |
| BMI (median, IQR) | 23.2 (19.6 - 26.2) | 25.17 (22.3 - 26.6) | 21.4 (20.3 - 22.8) |
| USA (n, %) | 3 (1%) | 4 (1%) | 2 (1.0%) |
| UK (n, %) | 155 (51.3%) | 244 (53.0%) | 90 (50.0%) |
| Canada (n, %) | 144 (47.6%) | 212 (46.1%) | 88 (48.9%) |
