# Supplementary figures and images for: Gut microbiome signatures reflect different subtypes of irritable bowel syndrome
Source: Gut Microbes. 2022 Dec 27;15(1):2157697. doi: 10.1080/19490976.2022.2157697 (PMC9809927; doi:10.1080/19490976.2022.2157697)

**A**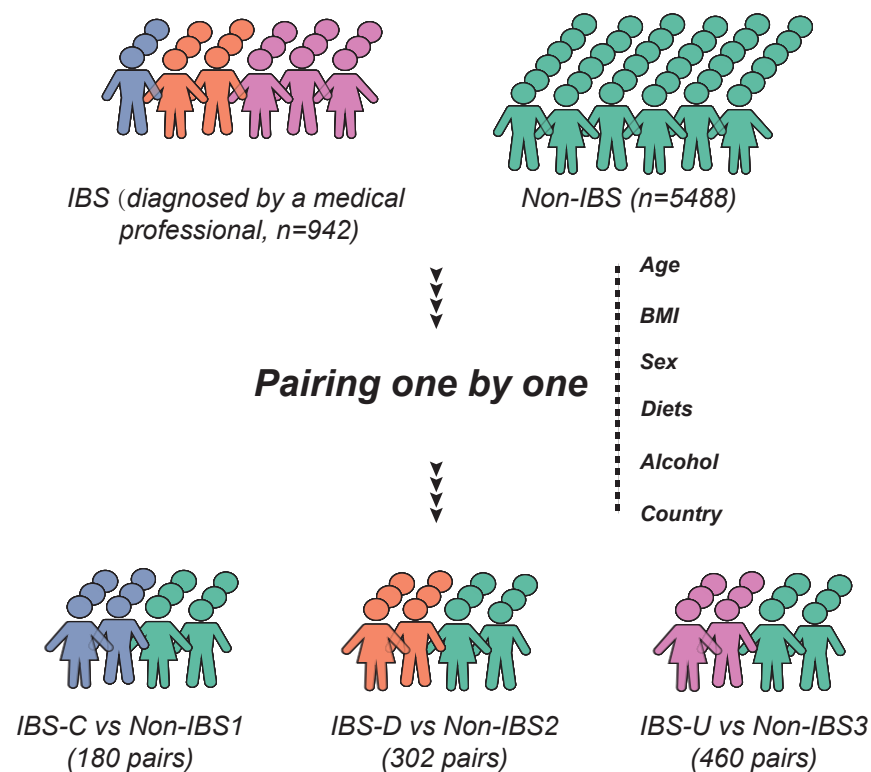**B**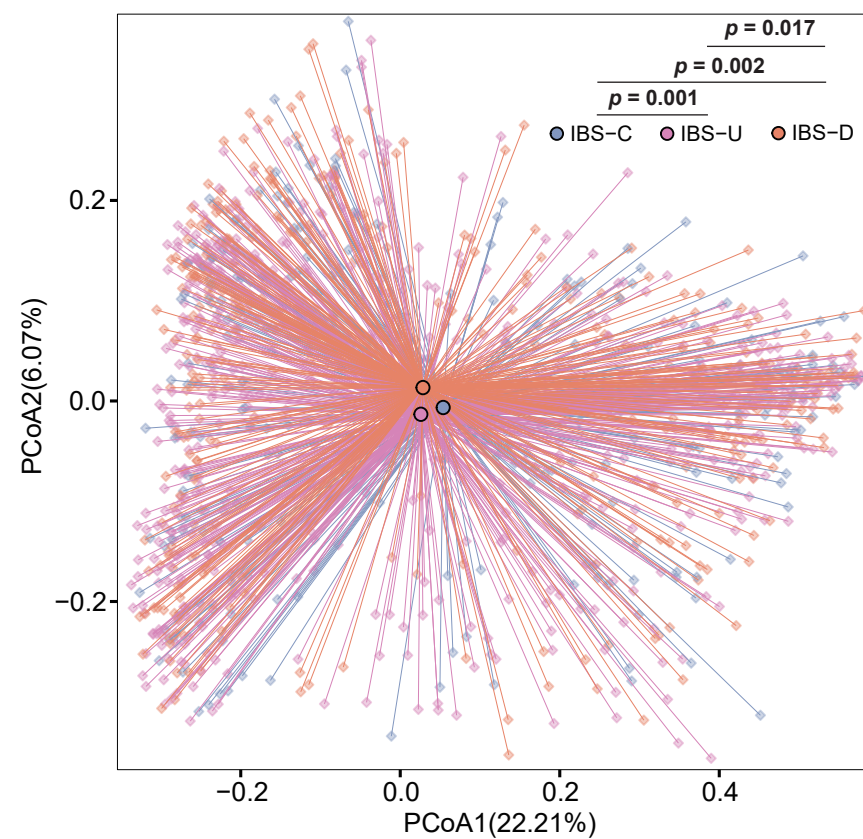**C**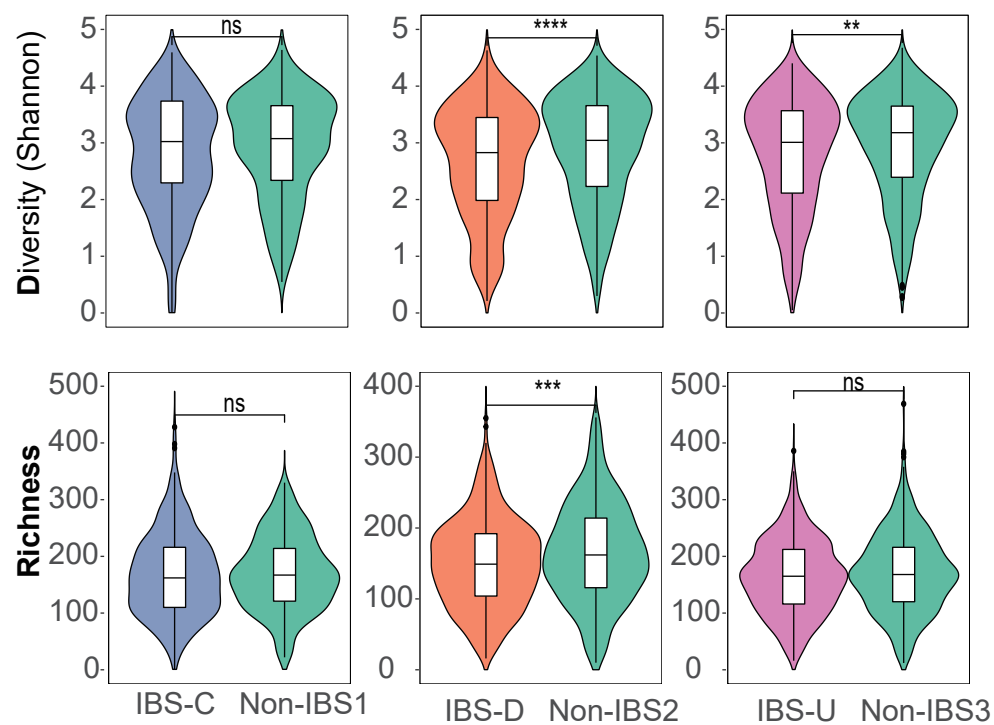**D**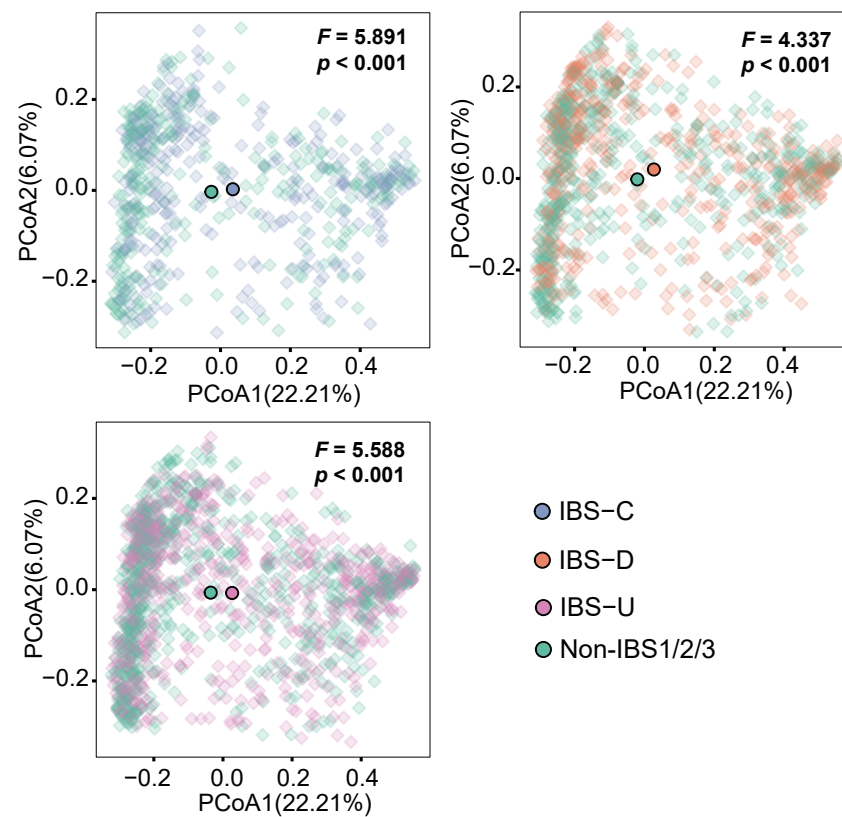

Supplement: Supplemental Material [file KGMI_A_2157697_SM8569.zip › Supplementary Figure 1.pdf]

A

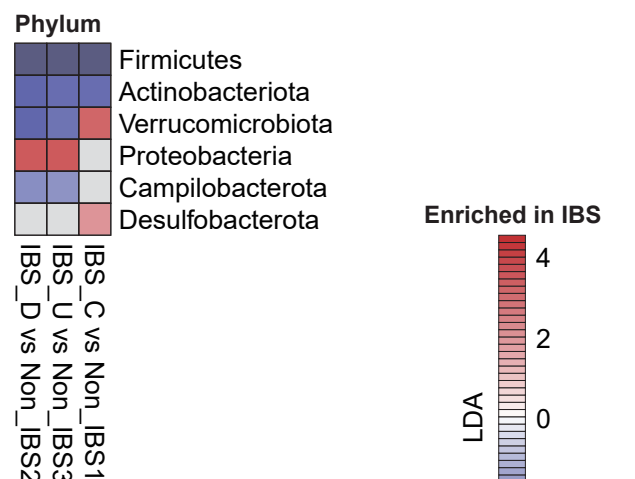

B

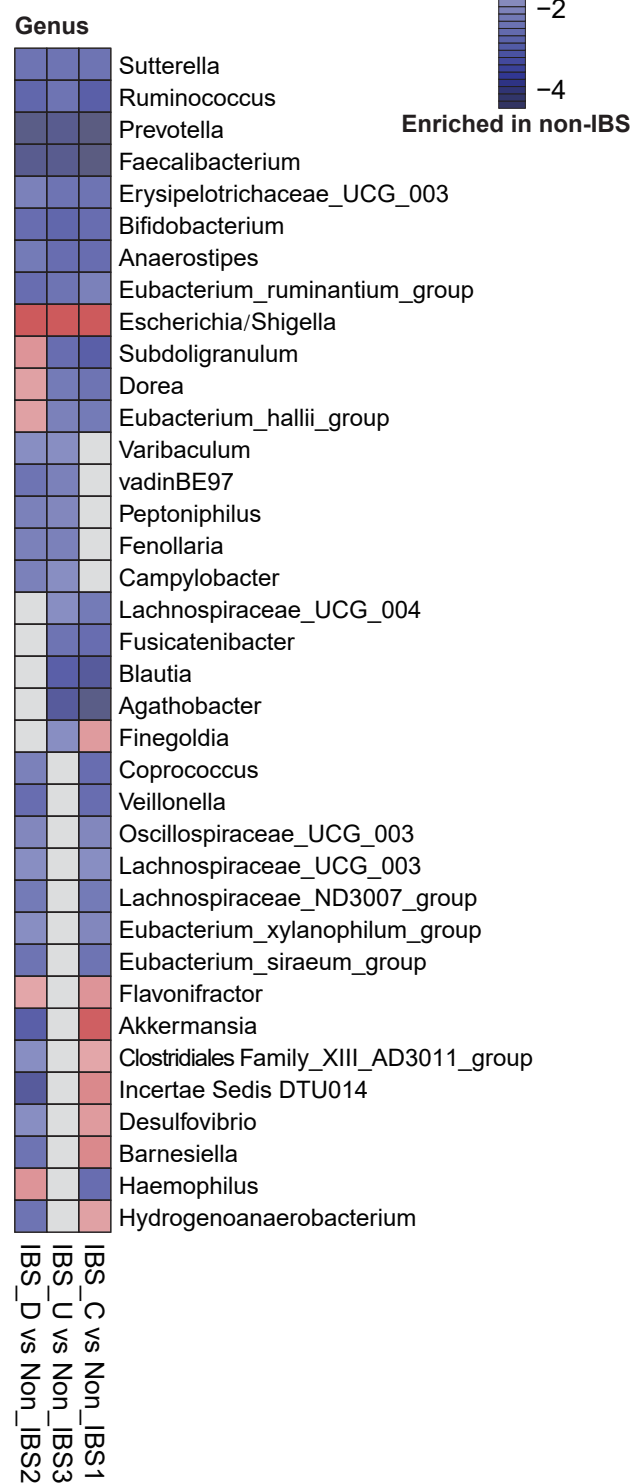

C

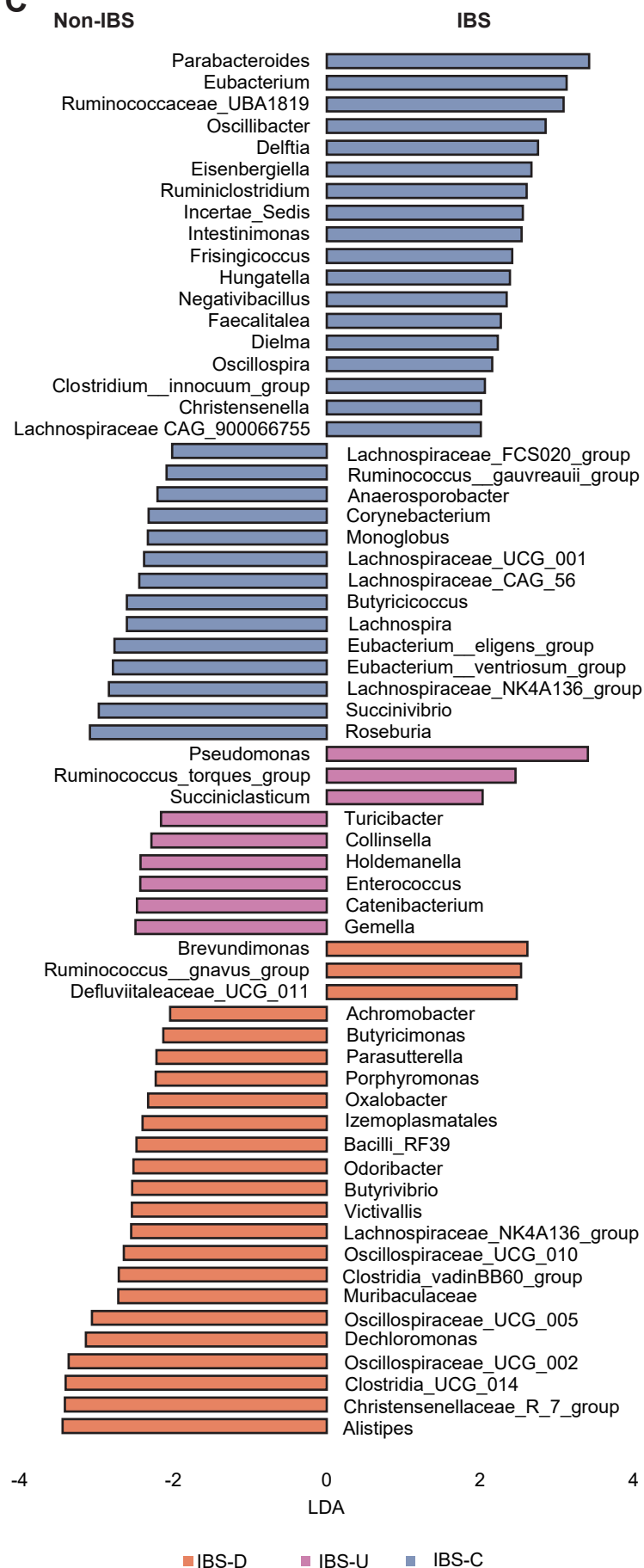

D

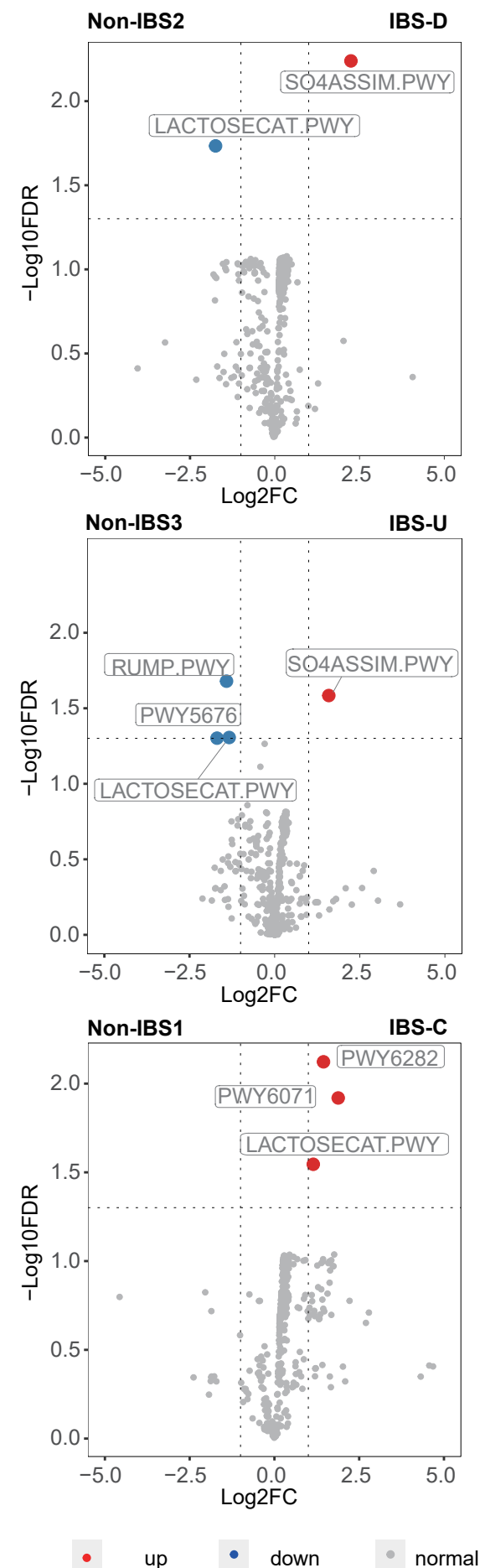

Supplement: Supplemental Material [file KGMI_A_2157697_SM8569.zip › Supplementary Figure 2.pdf]

**A**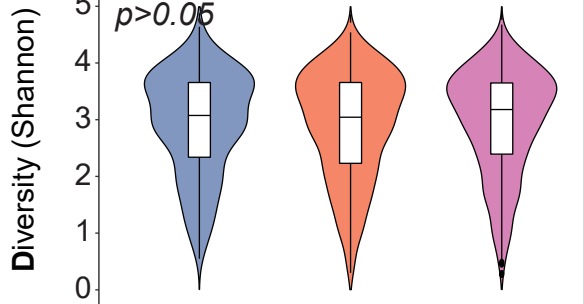**B**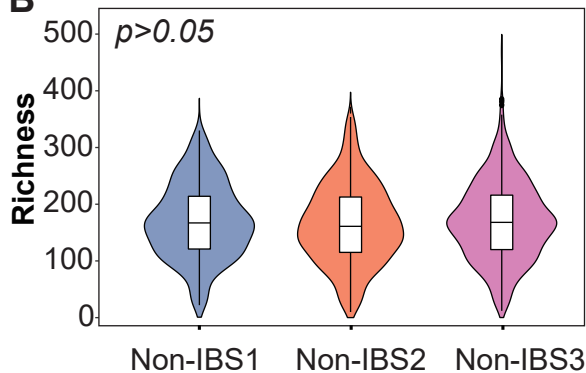**C**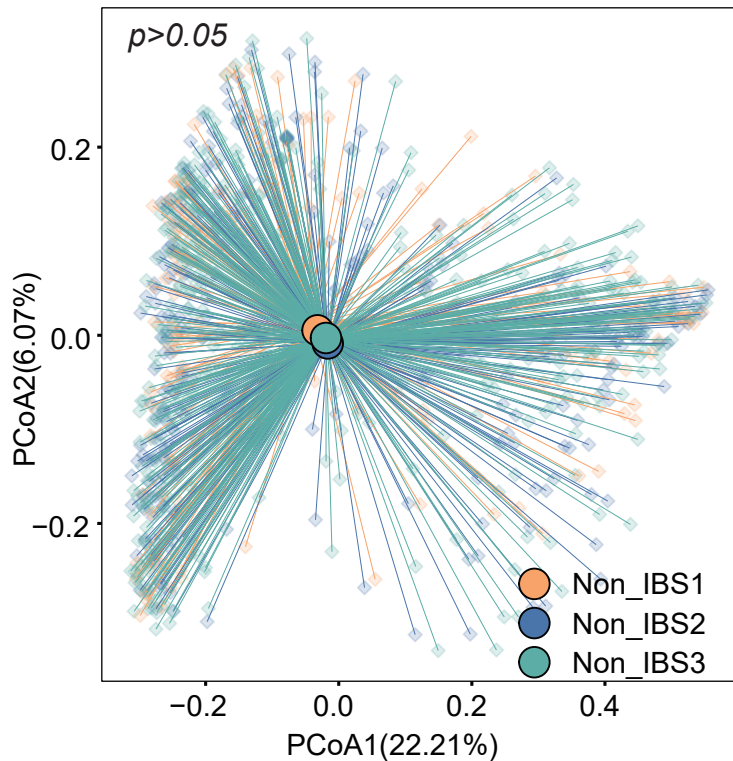

Supplement: Supplemental Material [file KGMI_A_2157697_SM8569.zip › Supplementary Figure 3.pdf]

**A**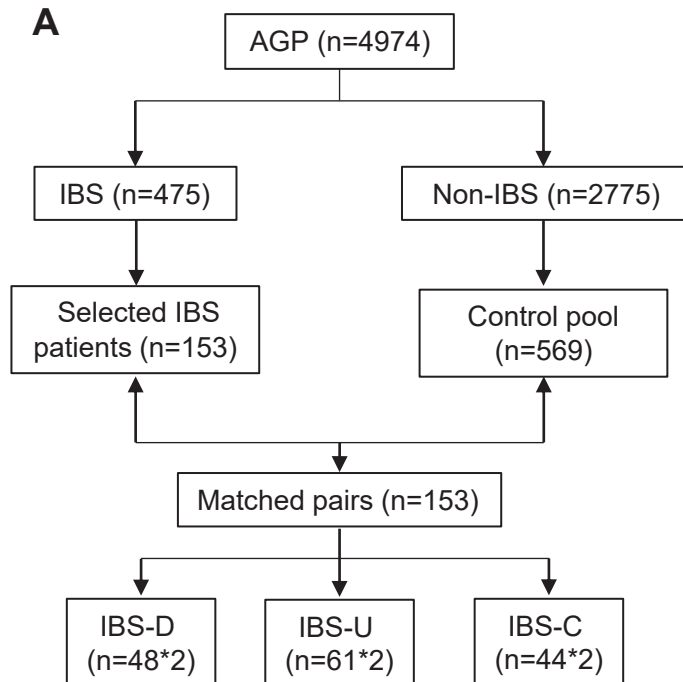**B**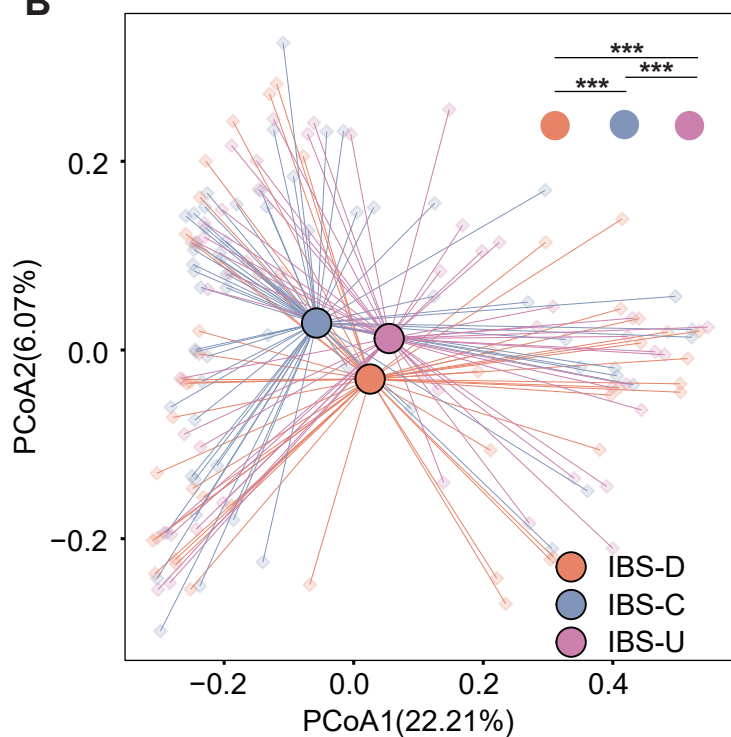**C**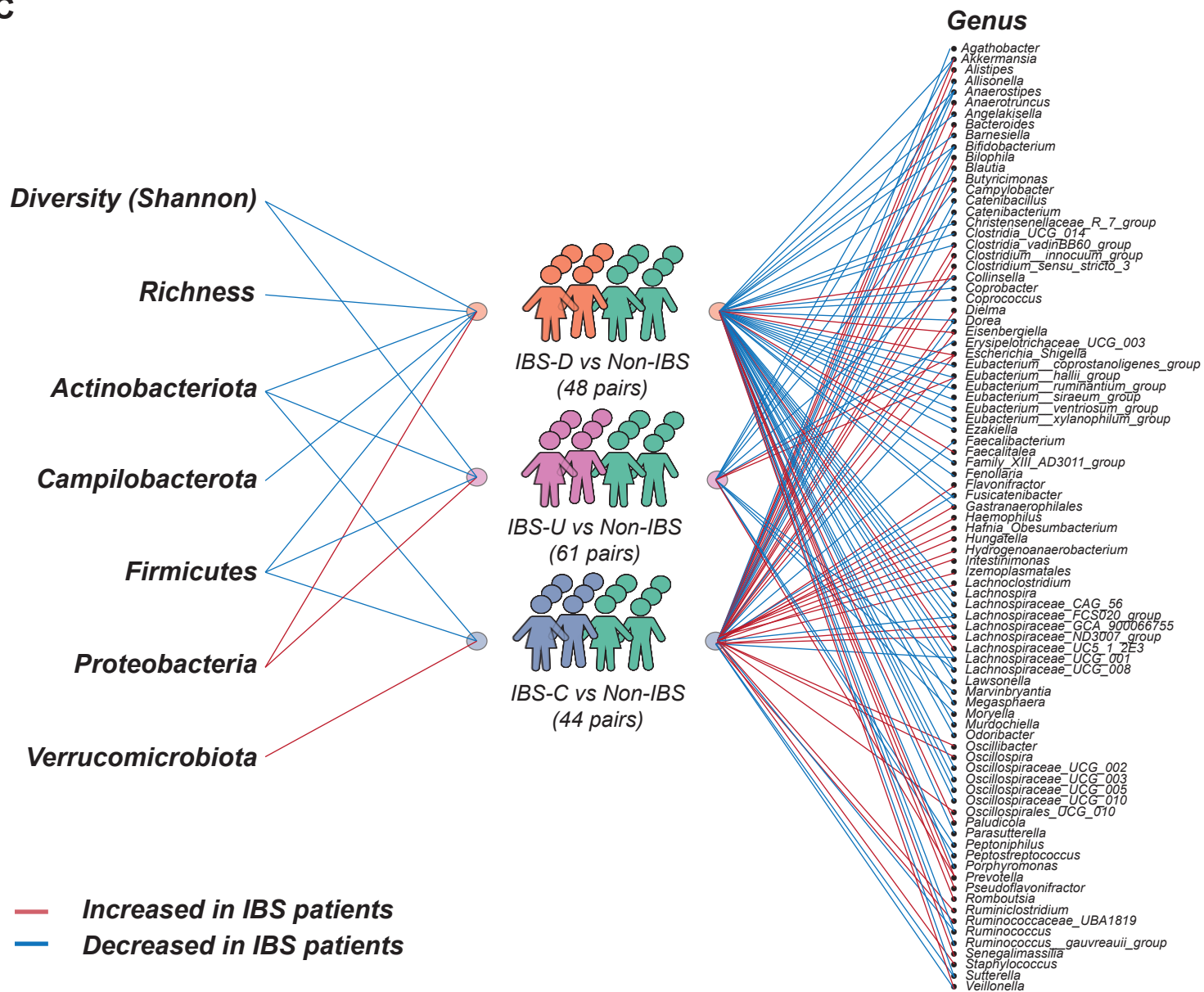

Supplement: Supplemental Material [file KGMI_A_2157697_SM8569.zip › Supplementary Figure 5.pdf]
