# Supplementary material for: Gut microbiome signatures reflect different subtypes of irritable bowel syndrome
Source: Gut Microbes. 2022 Dec 27;15(1):2157697. doi: 10.1080/19490976.2022.2157697 (PMC9809927; doi:10.1080/19490976.2022.2157697)
Supplement: Supplemental Material [file Supplementary Figure 6.pdf › Supplementary Figure 6.pdf]

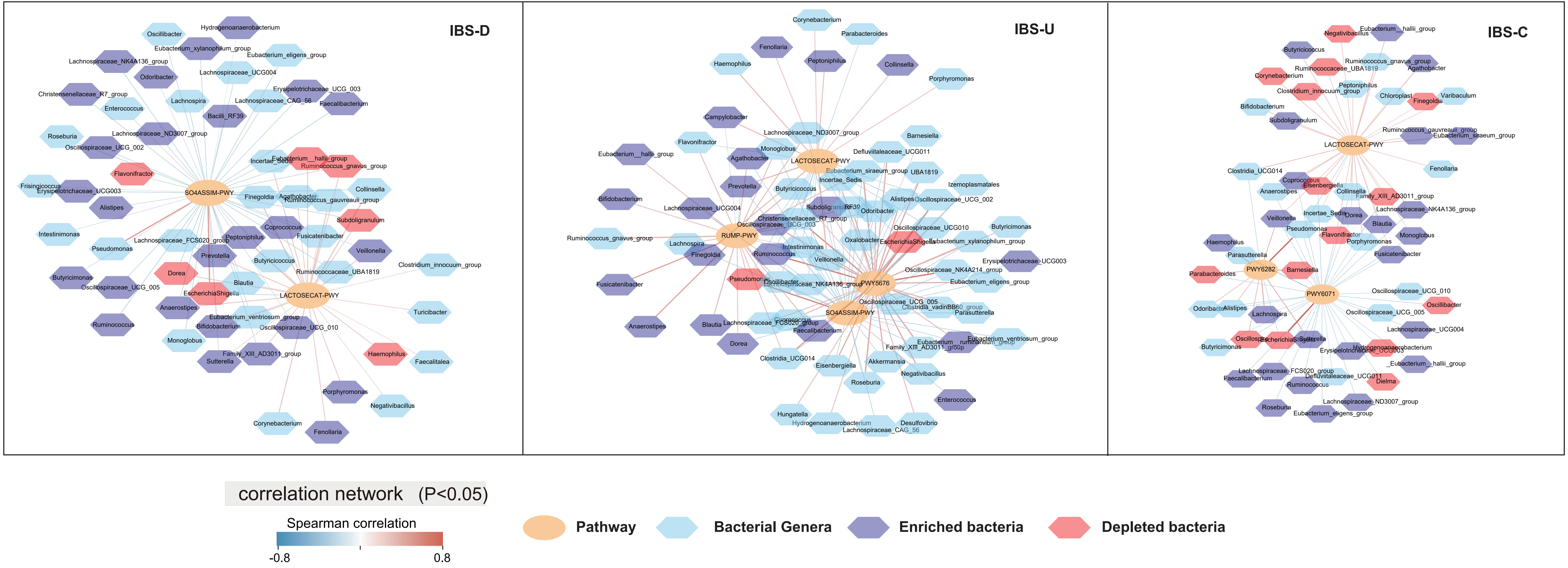

IBS-D
IBS-U
IBS-C
SO4ASSIM-PWY
LACTOSECAT-PWY
RUMP-PWY
PWY5676
PWY6282
PWY6071
correlation network (P<0.05)
Spearman correlation
-0.8
0.8
Pathway
Bacterial Genera
Enriched bacteria
Depleted bacteria
